# Multi-Faceted Role of Cancer-Associated Adipocytes in Colorectal Cancer

**DOI:** 10.3390/biomedicines11092401

**Published:** 2023-08-28

**Authors:** Adriana Grigoraș, Cornelia Amalinei

**Affiliations:** 1 Department of Morphofunctional Sciences I, “Grigore T. Popa” University of Medicine and Pharmacy, 700115 Iasi, Romania; 2 Department of Histopathology, Institute of Legal Medicine, 700455 Iasi, Romania

**Keywords:** cancer-associated adipocytes, tumor microenvironment, colorectal cancer, therapy

## Abstract

Colorectal cancer (CRC) is one of the most commonly diagnosed types of cancer, especially in obese patients, and the second cause of cancer-related death worldwide. Based on these data, extensive research has been performed over the last decades to decipher the pivotal role of the tumor microenvironment (TME) and its cellular and molecular components in CRC development and progression. In this regard, substantial progress has been made in the identification of cancer-associated adipocytes’ (CAAs) characteristics, considering their active role in the CCR tumor niche, by releasing a panel of metabolites, growth factors, and inflammatory adipokines, which assist the cancer cells’ development. Disposed in the tumor invasion front, CAAs exhibit a fibroblastic-like phenotype and establish a bidirectional molecular dialogue with colorectal tumor cells, which leads to functional changes in both cell types and contributes to tumor progression. CAAs also modulate the antitumor immune cells’ response and promote metabolic reprogramming and chemotherapeutic resistance in colon cancer cells. This review aims to report recent cumulative data regarding the molecular mechanisms of CAAs’ differentiation and their activity spectrum in the TME of CRC. A better understanding of CAAs and the molecular interplay between CAAs and tumor cells will provide insights into tumor biology and may open the perspective of new therapeutic opportunities in CRC patients.

## 1. Introduction

According to the World Health Organization (WHO), colorectal cancer (CRC) is the third most common cancer worldwide, after female breast cancer and lung cancer [1,2]. Moreover, it represents the second leading cause of cancer death, after lung cancer, with a continuous increase in incidence in the past decades, especially in high or very high Human Development Index (HDI) countries [1,2]. In this regard, over 1.9 million new cases and 915,880 deaths related to CRCs have been reported worldwide in 2020 [1,2], and these figures are expected to increase to over 3.2 million new cases and 1.6 million CRC-related deaths in 2040 [3]. Numerous studies showed a strong association between obesity, smoking, alcohol consumption, sedentary lifestyle, low-fiber and high-fat diet and CRC development [1,4,5]. Moreover, genetic factors, such as familial adenomatous polyposis, Lynch syndrome, and several gene mutations, such as adenomatous polyposis coli (*APC*), mothers against decapentaplegic homolog 4 (*SMAD4*), Kirsten rat sarcoma viral oncogene homolog (*KRAS*), cellular tumor antigen p53 (*TP53*), DNA mismatch repair protein Mlh1 (*MLH1*), and DNA mismatch repair protein Mlh2 (*MSH2*) have been registered in most cases [1]. In addition, inflammatory bowel disease, diabetes mellitus, gut microbiota composition, and drug use are also considered additional risk factors for colorectal carcinogenesis [1].

Taking into consideration these data, recent advances in tumor biology have demonstrated that the complex molecular interaction between tumor cells and their microenvironment plays an essential role in clonal selection, tumor cell heterogeneity, neovascularization, drug therapy resistance, and metastasis [4,5]. The CRC tumor microenvironment (TME) is a heterogeneous milieu, consisting of colorectal tumor cells, cancer stem cells, and extracellular matrix components (collagen, laminin, elastin, and fibronectin) associated with a spectrum of nontumor cells, which include cancer-associated fibroblast (CAFs), endothelial cells, cancer-associated adipocytes (CAAs), pericytes, mesenchymal stromal cells, and immune cells [6]. Additionally, the complex intercellular dialogue is supported by several molecules released in the tumor niche by the tumor and nontumor-associated cells, including growth factors, cytokines, adipokines, chemokines, and exosomes [7].

CRC can be currently stratified into four Consensus Molecular Subtypes (CMS1-4) with distinguishing features, according to the TME dynamic composition and different tumor gene expression. CMS1 displays an immune system signature, along with marked B-Raf proto-oncogene and serine/threonine kinas (*BRAF*) mutations, while CMS2 is an epithelial-like type CRC (canonical type), characterized by c-Myc and Wnt signaling axis activation. In an analogous manner with CMS2, CMS3 displays an epithelial-like feature, being characterized by metabolic dysregulation and *KRAS* mutations. CMS4 exhibits a mesenchymal phenotype, being characterized by increased stromal invasion, prominent inflammation, angiogenesis, and tumor growth factor β (TGF-β) activation [8]. 

Significant epidemiological data have been reported regarding the association between CRC and obesity, as well as the role of visceral adipocytes and CAAs, in colorectal carcinogenesis and metastasis [1,9]. CAAs have been described by Dirat et al., as modified characteristic adipocytes with a specific biological activity in a co-culture of 3T3-F442A mature adipocytes with breast tumor cells, in an in vitro study [10]. From this moment, their potential role in carcinogenesis and tumor growth has been explored in different types of tumors, including breast, colon, ovarian, or pancreatic cancers [5,11,12]. Despite the intensive effort dedicated to CRC research, the molecular “dialogue” between CAAs and colorectal cancer cells in the tumor niche remains only partially known [5,11]. In this context, this review summarizes the most recent knowledge regarding CAAs differentiation, along with their involvement in colorectal carcinogenesis, in order to develop new therapeutic approaches that would counteract tumor progression.

## 2. The Crosslink between Obesity and Colorectal Cancer

Obesity, defined by the World Health Organization (WHO) as a body mass index (BMI) ≥ 30 kg/m^2^ in adult persons, is one of the main factors in colorectal carcinogenesis, along with several factors, including smoking, low-fiber diet, and sedentary lifestyle [13,14]. The positive correlation between obesity and CRC is supported by different meta-analyses, which have revealed that a body weight gain of 10 Kg is associated with an 8% increased risk of CRC development [15,16]. Moreover, according to recent data, obese persons have a 1.3-fold higher risk for CRC development than nonobese persons, especially men [17,18], and an increase in the waist circumference by 2 cm is accompanied by a 4% higher risk of CRC development [19]. In addition to these findings, the results of a study performed on a large population-based cohort also noticed a decrease in the overall survival rate in CRC cases in patients with a BMI between 25 and 50 kg/m^2^ compared to CRC patients with a BMI lower than 25 kg/m^2^ [19]. On the other hand, body weight loss after bariatric surgery decreases the colorectal carcinogenesis risk by 27% [20]. 

Although obesity plays a crucial role in colorectal carcinogenesis, the molecular obesity-driven mechanisms that support CRC development are still partially unknown. 

Adipose tissue includes three different types of cells (white, brown, and beige adipocytes), which are grouped into four types of fat tissues, namely white (WAT), brown (BAT), beige, and perivascular adipose tissue (PVAT) [21]. WAT is divided into subcutaneous adipose tissue (SAT) and visceral adipose tissue (VAT). VAT is mainly composed of white adipocytes, associated with several other types of cells, such as preadipocytes, pericytes, endothelial cells, and immune cells [22]. VAT is currently considered to be involved in different pathological conditions, such as cardiovascular diseases or diabetes mellitus [23,24]. Due to its proximity to the gastrointestinal tract, VAT contributes to the TME proinflammatory profile by releasing a panel of factors, including growth factors, hormones, proinflammatory cytokines, and chemokines, including interleukin 1 (IL-1), interleukin 6 (IL-6), interleukin 12 (Il-12), tumor necrosis factor (TNF-α), leptin, ghrelin, resistin, and adiponectin, which promote colorectal carcinogenesis in obese people [1]. Despite the protumorigenic action of these adipokines, it remains to be determined whether they support CRC development by a direct effect on colorectal mucosal cells or by a paracrine manner that induces a TME proinflammatory milieu [25]. 

Mainly studied in breast cancer, VAT adipocytes surrounding the colorectal tumor cells display a different phenotype and are called CAAs [4]. CAAs have also been described in other types of cancers, including colon, prostate, ovarian, and pancreatic tumors [26,27]. Although CAAs are partially studied in CRC, they may play a relevant role in colorectal carcinogenesis, based on the complex molecular dialogue between CAAs and colorectal tumors taking place in the TME.

## 3. Cancer-Associated Adipocytes (CAAs) Characteristics

The mature adipocytes disposed near the tumor invasive front can be transformed into CAAs, as a consequence of their close relationship with tumor cells, adopting a fibroblast-like phenotype and releasing a panel of adipokines and proinflammatory cytokines that promote cancer progression and metastasis [28]. Although traditionally considered as peritumoral white adipocytes, CAAs or reactive adipocytes are now considered one of the main TME components that promote neoangiogenesis, tumor growth, metastasis, and drug therapy resistance [5]. 

**Figure 1:**
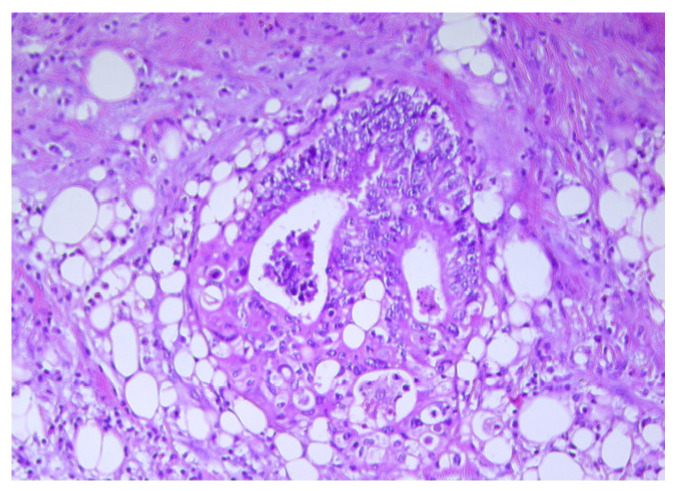
Numerous small size adipocytes disposed at the tumor invasive front in a colon carcinoma, H&E ×20.

**Figure 2:**
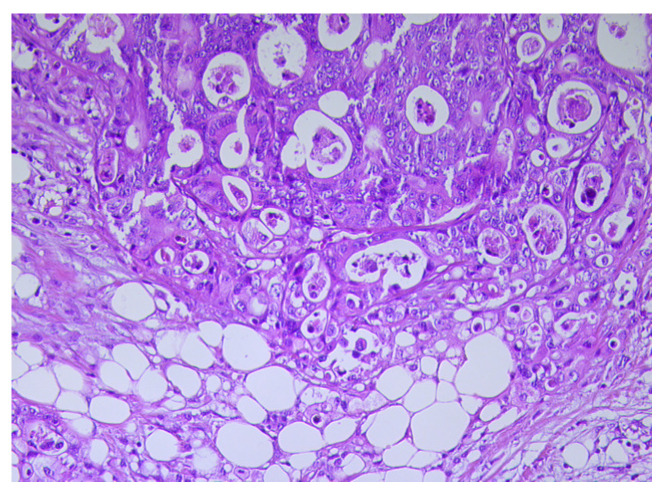
Tumor cells and adipocytes with different sizes in a colon carcinoma, H&E ×20.

Compared to mature adipocytes, CAAs display a small size and fibroblast-like features (Figure 1 and Figure 2) [5]. In addition, unlike the mature adipocytes located at a distance from the cancer invasion front, CAAs possess a high proliferation index and strong migratory capacity [29]. CAAs also exhibit a decrease in their triglyceride content due to disturbance of the lipid storage capacity associated with lipoprotein lipase (*LPL*) and fatty acid synthase (*FAS*) gene expression downregulation, as observed in surgically treated CRC patients [26,28]. These data are supported by the results of different studies focused on tumor progression in several types of cancers, including malignant melanoma, breast, colorectal, gastric, and renal cancers [5,28]. According to these data, the mature white adipocytes are replaced with fibroblast-like cells without lipid droplets content in the cytoplasm and a desmoplastic stroma appears when the cancer cells infiltrate the surrounding adipose tissue [30,31,32]. 

CAAs also exhibit numerous mitochondria and a decreased expression of hormone-sensitive lipase (HSL), an intracellular neutral lipase that regulates the cytoplasmic fat storage [5,28]. 

Moreover, CAAs have a decreased expression of fatty acid binding protein 4 (FABP4), a terminal adipocyte differentiation marker, which regulates the peroxisome proliferator-activated receptor gamma (PPARγ) expression [5,28,29,33,34]. Additionally, CAAs display a reduced expression of other terminal differentiation markers, including CCAAT-enhancer-binding proteins alpha (C/EBPα) and adiponectin, while the protumorigenic factors, such as resistin and leptin, are overexpressed [29]. 

Due to the adoption of a fibroblast-like morphology, CAAs also have an overexpression of fibroblast markers, including collagen I, α-smooth muscle actin (α-SMA), and matrix metalloproteinase 11 (MMP11) [35]. 

PD-L1 increased expression has been additionally identified in CAAs, a feature which supports their tumoral immunosuppression role in the TME. Moreover, PD-L1 overexpression is also observed in brown adipose tissue, suggesting that CAAs have browning features [28]. In addition, a programmed death-ligand 1 (PDL-1)-mediated inhibition of the antitumor activity of CD4+ and CD8+ T cells has been noted in breast cancers and CRC due to their metabolic activity modulation [5,28]. Thus, the downregulation of PD-L1 expression in peritumoral adipose cells may lead to an improvement in the antitumor activity of anti-PD-L1 or anti-PD-1 antibodies [5].

It has been recently suggested that CAAs may display senescent features, mainly in obese patients [28]. In this regard, the upregulation of genes associated with the cell cycle arrest accompanied by an increased expression of tumor-suppressive pathways have been registered in breast cancer CAAs [28]. Furthermore, autophagy induced by Cav-1, an integral membrane protein, might represent a significant correlation between CAAs and cellular senescence [28]. However, according to our knowledge, these features related to the activation of many oncogenes associated with CAAs senescent-like phenotype have not been reported in CRC until now.

## 4. Potential Mechanisms of CAAs Differentiation in CRC 

The peritumoral adipocytes represent a significant source of the energy for proliferating cancer cells. Although numerous studies have certified the presence of CAAs in colorectal tumors, the underlying mechanism associated with their origin is only partially deciphered [5,36,37]. In advanced stages of CRC cases, the tumor mass extends into the serous layer of the intestine and establishes direct contact with the white adipose cells usually present in the peritoneum [36]. Additionally, the white adipocytes presence has been registered in the submucosal layer of patients with bowel disease and CRC, leading to the conclusion that the adipocytes are part of the TME even from the early CRC stages [1,38].

**Figure 3:**
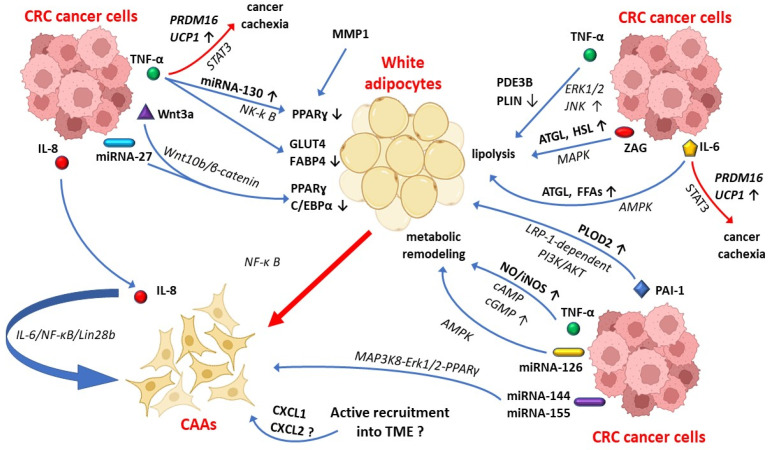
The mechanisms involved in CAAs differentiation in CRC. Tumor-derived Il-6 activates the lipolysis cascade in peritumoral white adipocytes through AMPK signaling pathway that increases the adipose triglyceride lipase (ATGL) and the free fatty acids (FFAs) release in TME. ZAG released by cancer cells stimulate lipid decomposition by ATGL and HSL-increased expression via MAPK signaling pathway. In addition, ERK1/2 and JNK mediated pathways induce PDE3B- and PLIN-decreased expression that promote CAAs differentiation. Tumor-derived TNF-α induces metabolic remodeling in adipose tissue by increasing iNOS/NO through cAMP/cGMP pathway signaling activation. PAI-1 activates LRP 1-dependent PI3K/AKT signaling axis and promotes the activation of PLOD2 in CAAs. Tumor-derived miRNAs are also involved in metabolic remodeling and CAAs differentiation through AMPK, NK-kB, and MAP3K8-Erk1/2-PPARγ signaling axis activation. Moreover, active recruitment of adipocytes by TME is another potential mechanism of CAAs differentiation in CRC. AMPK—AMP-activated protein kinase; ATGL—adipose triglyceride lipase; CAAs—cancer-associated adipocytes; C/EBPα: CCAAT-enhancer-binding protein α; cAMP—cyclic adenosine monophosphate; cGMP—cyclic guanosine monophosphate; CRC—colorectal carcinoma; CXCL—chemokine (C-X-C motif) ligand; ERK1/2—Ras-dependent extracellular signal-regulated kinases 1/2; FABP4—fatty acid binding protein 4; FFAs—free fatty acid; GLUT4—insulin-responsive glucose transporter 4; HSL—hormone-sensitive lipase; JNK—c-Jun N-terminal kinase; IL-6—Interleukin 6; IL-8—interleukin 8; iNOS—nitric oxide synthase; MAPK—mitogen-activated protein kinases; miRNA—microRNA; MMP11—matrix metalloproteinase 11; NO—nitric oxide; PAI-1—plasminogen activator inhibitor type 1; NK-kB—Nuclear factor-κB; PDE3B—phosphodiesterase 3B; PI3K/AKT—phosphatidylinositol 3-kinase/protein kinase B; PLIN—lipid droplet-associated protein; PLOD2—procollagen-Lysine,2-oxoglutarate 5-dioxygenase 2; PPARɣ—peroxisome proliferator-activated receptor gamma; PRDM16—PR domain zinc finger protein 16; STAT3—signal transducer and activator of transcription 3; TME—tumor microenvironment; TNF-α—tumor necrosis factor α; UCP1—uncoupling protein1; Wnt/-catenin—wingless-type MMTV integration site family/-catenin signaling pathways; ZAG—zinc-α2-glycoprotein; ↑—increase; ↓—decrease; ?—working hypothesis.

Currently, it is considered that cancer stem cells and cancer cells are able to induce the dedifferentiation of the neighboring adipocytes into CAAs, through the secretion of numerous factors, which act as a paracrine and autocrine signal on peritumoral adipocytes [5,37]. In this regard, recent research has shown that colon cancer cells release a spectrum of factors into the TME, including TNF-α, interleukin-6 (IL-6), interleukin-8 (IL-8), plasminogen activator inhibitor-1 (PAI-1), MMP1, and tumor-derived miRNAs that may contribute to this process (Figure 3) [1,29]. 

TNF-α released by tumor cells promotes the peritumoral adipocytes’ transformation into CAAs, through overexpression of miRNA-130, which decreases the transcription of PPARγ and promotes the adipocytes dedifferentiation by NF-κB pathway activation [29]. TNF-α exposure also prevents the dedifferentiation of 3T3-Ll adipocytes into mature adipocytes, through downregulation of gene expression of terminal differentiation markers, such as insulin-responsive glucose transporter 4 (GLUT4) and FABP4 [39]. In addition, the downregulation of PPARγ and C/EBPα in peritumoral fat cells is also induced by the activation of Wnt10b/β-catenin pathway signaling that contributes to their transformation into CAAs [29,40]. These data are supported by the results of research performed by Gustafson and Smith, who showed that 3T3-L1 adipocytes exposed to Wnt3a induce the CAAs phenotype occurrence, by increasing the expression of the undifferentiated adipocytes markers, such as Wnt10b, Pref-1/Dlk1, and Gata2 [41]. According to the presented data, since CAAs display progenitor-like features, characterized by lack of adipocyte terminal differentiation markers, it is considered that CAAs in CRC may occur from a process of dedifferentiation of mature adipocytes towards a progenitor-like status [1].

On the other hand, TNF-α supports the metabolic remodeling of peritumoral adipocytes, by increasing the nitric oxide (NO) and nitric oxide synthase (iNOS) via cAMP/cGMP pathway signaling activation [29]. Additionally, PDE3B and PLIN decreased expression via ERK1/2 and JNK pathways axis occurs in the TNF-α-rich TME that leads to CAAs lipolysis activation [29]. The lipolysis cascade activation in peritumoral adipocytes is also promoted by tumor-derived IL-6 via the AMPK pathway signaling that increases the adipose triglyceride lipase (ATGL) and the free fatty acid (FFAs) release in the TME [29]. IL-6 also leads to lipid adipocytes decomposition though overexpression of SOCS3 mediated by STAT3 pathway signaling [42]. 

The peritumoral adipocyte dedifferentiation in CRC is also supported by Zinc-α2-glycoprotein (ZAG) released by cancer cells that increases the lipolysis to ATGL and hormone-sensitive lipase (HSL) activation through MAPK signaling axis (Figure 3) [29,37]. MMP1 is another factor that induces the reduction in the size and number of lipid droplets and contributes to the dedifferentiation of the peritumoral adipocytes into CAAs by PPARγ downregulation [29]. Taken together, the peritumoral adipocytes undergo profound phenotypic adaptation characterized by lipid droplets depletion due to the close interaction with CCR cancer cells and their molecular factors released into the TME. 

PAI-1 is a potential modulator released by tumor cells and nontumoral stromal cells (e.g., endothelial cells and macrophages), which promotes tumor angiogenesis and supports cancer cells survival and resistance to chemotherapy-induced apoptosis [43,44]. PAI-1 may also contribute to reprogramming of peritumoral adipocytes toward CAAs, by activation of PLOD2 via LRP-1-dependent PI3K/AKT signaling axis [29]. In addition, PAI-1 stimulation leads to blockage of αvβ3-mediated cell migration on vitronectin and of tumoral extracellular matrix remodeling, which support the cancer cell migration in several types of cancers, including CRC [29,44]. 

In addition, a redifferentiation process of CAAs into other cell lineages has been described in the TME [45]. As a result, CAAs possess increased plasticity induced by tumor cells and they are able to transform into other cancer-supportive cells [35].

Tumor-derived miRNAs represent another suitable mechanism for CAAs differentiation (Figure 3) [35]. In this regard, cancer-derived miRNA-27 may suppress PPARγ and C/EBPα expression and may induce their change of phenotype into CAAs, in CRC cases [29,46], while a miRNA-27b-3p overexpression has been accompanied by an inhibition of browning in WAT [47]. In addition to these findings, recent studies revealed that tumor cells release miRNA-126, which induces metabolism remodeling in the peritumoral adipocytes through AMPK pathway activation and disruption of the IRS/Glut-4 axis [48,49].

Another possible mechanism of CAAs differentiation in CRC could be represented by the active recruitment of adipocytes by the TME [37]. This process has been registered in pancreatic cancer, where adipocytes can migrate into the TME under CXCL1 and CXCL2 signaling [50]. According to the current literature data, the active migration of adipocytes into the TME has not been reported in CRC cases [37]. In addition, there is not enough data related to the possible molecular factors involved in this process in CRC patients [37].

Furthermore, recent studies indicate that TNF-α and IL-6 promote cancer cachexia in different types of cancer, including gastric and CRC, by protein 16 containing PR domain (*PRDM16*) gene overexpression via the STAT3 signaling pathway, which induces the browning of WAT [29,37,51]. In this regard, CAAs are considered to arise along with beige differentiation or WAT browning in CRC patients, especially in advanced stages [28,52]. In addition, cancer-derived miRNA-144 and miRNA-155 may modulate the beige/brown adipocytes differentiation of CAAs through MAP3K8-Erk1/2-PPARγ signaling pathway [35]. These data are supported by current research, which has shown that IL-6 increases the uncoupling protein 1 (UCP1) expression in WAT, in gastric and CRC patients, with UCP1-positive cells being mainly detected in advanced cancer stages [52]. Moreover, according to the same research, IL-6 induces cancer cachexia through regulation of WAT lipolysis in early-stage and browning of WAT in late-stage cancer cachexia [52]. Although the molecular mechanism that leads to WAT browning related to cellular components of the TME is incompletely disentangled in CRC patients, it is considered that TNF-α, IL-1, IL-6, IL-8, and C-X-C motif chemokine ligand 8 (CXCL8) are the most relevant procachectic molecules, which are partially produced by tumor cells, CAAs, and immune-associated tumor cells [37]. 

As part of the TME, CAAs secrete IL-8 which contributes to their maintenance in an activated status via IL-6/NF-κB/Lin28b feedback-loop activation [29]. In addition, IL-8 is one of the most significant activating chemokine of cancer-associated cells, along with TNF-α and IL-6, which contribute to the proinflammatory profile of the TME in CRC patients [37,53]. IL-8 induces the activation of JAK/STAT3 oncogenic signaling cascade that supports CRC cell proliferation, tumor angiogenesis, and metastasis [54,55,56]. 

Taking into consideration this accumulated data, a deeper understanding of the molecular mechanisms that induce CAAs differentiation in the TME will open the perspective for designing novel therapeutic strategies in CRC.

## 5. The CAAs Role in CRC Progression

### 5.1. CAAs Adipokines in CRC

Obesity and VAT have a significant impact on the overall survival of many types of cancers, including CRC. The tumor cells surrounding adipocytes release a spectrum of adipocytes-derived factors, which induce local inflammatory cell recruitment and create a TME able to support the tumor growth in CRC patients. Since 1994, the moment of leptin identification, more than 400 adipocytes-derived factors have been described [57]. The most important adipokines, with impact in colorectal carcinogenesis, released by CAAs are leptin, adiponectin, apelin, ghrelin, resistin, visfatin, insulin, insulin-like growth factors (IGFs), vascular endothelial growth factor (VEGF), interleukins (IL-6, IL-8, and IL-10), inflammatory cytokines (TNF-α and PAI-1), and chemokine (C-C motif) ligand 2 (CCL2) [14,36,58].

Leptin is a hormone known to be involved in the energy-balance regulation mainly released by adipocytes [59]. Different studies have highlighted an increased leptin level in obese CRC patients due to the increased leptin resistance, while soluble leptin receptor (sOB-R) circulating level has been observed to be inversely proportional with the risk of CRC development [60,61,62]. Leptin binds to its specific receptor, OB-R, and induces IL-6, MMPs, and TGF-β overexpression by c-Jun, Akt, and JAK/STAT3 signaling pathways activation, which promote colorectal carcinogenesis (Table 1) [5,14]. In addition, leptin is able to support the lamellipodia formation and tumor cell invasion through activation of cell division control protein 42 (Cdc42) and Ras-related C3 botulinum toxin substrate 1 (Rac1) in human tumor colorectal cell lines LS174T and HM7 [63].

Adiponectin is an adipokine that contributes to nutrient homeostasis, being balanced by leptin. Adiponectin also acts as an anticancer factor through the inhibition of tumor cell proliferation, angiogenesis, and metastasis via AMPK activation [5,64,65]. Adiponectin is also reducing the chronic inflammation induced in colon cancer by preventing the goblet cell apoptosis and by inducing the epithelial to goblet cell differentiation [66]. Considering the decreased plasma level of adiponectin detected in obese patients, it is considered a risk factor for CRC carcinogenesis [67,68,69]. These data are supported by a recent study that revealed decreased tumor cell survival and migration induced by adiponectin in CaCo-2 and HCT116 human colorectal cell lines [70]. According to the same study, adiponectin and adiponectin receptor levels represent potential biomarkers used for CRC survival assessment [70]. Moreover, a positive correlation between the tumor size and adiponectin receptor 1 (AdipoR1) expression has been registered in the early stages of CRC cases [71], while its overexpression is also associated with lymph node metastasis and poor prognosis [72]. In addition, an immunohistochemical study performed on 104 newly diagnosed CRC patients showed that adiponectin receptor 2 (AdipoR2) had a higher association with lymph node metastases compared with AdipoR1 [73]. Based on these observations, the complex involvement of adiponectin–leptin imbalance in CRC growth needs future investigation and may lead to identification of new therapeutic strategies in these patients. 

Apelin is an adipokine associated with signaling pathways involved in tumor growth and migration in different types of cancers [74]. Recently, apelin and its receptor have been identified as potential molecular factors correlated with a higher risk of lymph node and distant metastasis in CRC patients [75]. Ghrelin is another adipokine with a significant impact on tumor progression, which acts as a proapoptotic factor. Currently, its relationship with colorectal carcinogenesis is described in several experimental studies [1], but results are still inconclusive. According to these, decreased ghrelin levels promote a proinflammatory TME that contribute to colorectal tumor growth [58]. 

Resistin is a polypeptide produced by macrophages, rather than by adipocytes, that supports the TME inflammatory status in several types of cancers, such as nonsmall cell lung cancers or breast tumors that have also been suggested to be linked to CRC progression [1]. Resistin induces the activation of the Toll-like receptor 4 on the colon cancer cells and the overexpression of some growth factors, adhesion molecules, and proinflammatory cytokines (e.g., IL-6 and TNF-α) that promote tumor angiogenesis and metastasis [1,76]. In addition, different studies have reported a correlation between high serum resistin levels, tumor grade and CRC prognosis [77,78]. However, resistin is currently considered more likely a tumor marker than a risk factor in CRC patients [76] and its role in colorectal carcinogenesis should be certified by future research.

Visfatin, such as leptin, is another adipokine that supports colorectal carcinogenesis through different signaling pathways activation, such as ERK1/2, p38 MAPK, PI3K/mTOR, JNK, and JAK/STAT, which promote cell proliferation and metastasis [79]. In addition, a recent study revealed that visfatin also acts as an inhibitor of the 5-fluorouracil (5-FU) therapeutic effect in CRC patients through visfatin/SDF-1/Akt pathway activation (Table 1) [80]. 

It has been demonstrated that the insulin/IGFs axis plays a significant role in colorectal carcinogenesis [81,82], by PI3K/Akt pathway activation, representing an essential therapeutic target axis in CRC patients [83]. In this regard, PI3K/Akt axis activation induced by insulin/IGFs has been observed to increase the resistance to 5-FU and cycloheximide cytotoxicity in HT29 colon cancer cell line [81]. In addition, a reduction in Src activity, a tyrosine protein kinase, which activates the PI3K/Akt pathway, leads to inhibition of tumor cell proliferation and metastasis in SW48 and HT-29 colon cancer cell lines [84]. In addition, it has been demonstrated that the insulin-like growth factor 1 (IGF1) and IGF1 receptor (IGF-1R) overexpression in colonic mucosae leads to Src activation that supports the proliferation of human colorectal carcinoma cell lines [85]. Moreover, more recent data revealed that the insulin receptor activation is a fundamental event in the early stages of colorectal tumorigenesis [86].

The molecular interplay between cancer-associated cells and colorectal cancer cells in the TME involves a spectrum of inflammatory cytokines. These are partially released by CAAs and include IL-6, IL-8, IL-10, TNF-α, PAI-1, and CCL2 [87]. Among them, IL-6 is one the most proinflammatory cytokines found in the TME, in both experimental and human colon cancer models, which has been associated with CRC progression and metastasis through activation of the STAT3 pathway axis in tumor cells [5,88,89]. The proinflammatory status of the TME is also associated with TNF-α that promotes colorectal carcinogenesis and tumor cell migration via NF-kB and Wnt/β-catenin signaling pathways activation [90,91]. Furthermore, enhanced cancer cells invasion and the metastasis ability of HCT-116 human colon cancer cells exposed to low doses of TNF-α has been demonstrated in a recent study [92]. This effect achieved via the ERK1/2 pathway axis leads to an overexpression of tumor-associated calcium signal transduction protein 2 (TROP-2), a transmembrane glycoprotein expressed in many types of human epithelial tumors, including CRC [92]. 

An increased PAI-1 serum level has been registered in CRC patients [14]. Although this value has been associated with an inflammatory reaction in the colonic epithelium, PAI-1 is not considered a relevant factor for colorectal carcinogenesis [93]. However, PAI-1 may induce colonic mucosae lesions through the upregulation of TGF-β via the PAI1–tPA axis in colitis [94]. 

CCL2 or monocyte chemoattractant protein 1 (MCP-1) is a key inflammatory cytokine of the TME that is overexpressed in obese CRC patients [14]. It stimulates the local recruitment of macrophages and is associated with tumor progression, induced by the inflammatory profile of the tumor niche in CRC cases [14]. The potential role of CCL2 in colorectal carcinogenesis has been demonstrated by a study on *Apc^Min/+^*/MCP-1^−/−^ mice model that revealed a reduction in tumor progression induced by local increased cytotoxic T lymphocyte and decreased regulatory T cells activity [95]. Consequently, the downregulation of CCL2 and TROP-2 expression may decrease the TME inflammatory status and reduces the tumor cells growth in CRC cases.

VEGF is a proangiogenic factor produced by CAAs, that is critical in CRC progression and metastasis (Table 1) [5]. Several studies have shown a direct correlation between serum and tissue VEFG expression and poor overall survival in CRC patients [96,97,98]. As a consequence, VEGF is currently considered one of the most relevant factors in new blood vessel formation, during the early and late CRC stages [96]. Moreover, the TME “mosaic vessels” exhibit an atypical morphology. They have an incomplete lining endothelium and, as a consequence, colon cancer cells are exposed to the lumen of these blood vessels [5]. Thus, due to these particular features, the TME vessels have a dual involvement in cancer progression, promoting tumor cell metastasis and interfering with uniform drug delivery into the CRC TME [1,5].

CAAs also have the role of promalignant stromal cells through extracellular tumoral matrix remodeling and by creating a tumor tolerant niche. Higher levels of tenascin, periostin, collagen, cytokines, chemokines, growth factors, exosomes, and matrix remodeling enzymes have been demonstrated to be included in the extracellular tumor matrix, being released by nontumoral stromal cells, including CAAs [5]. In this regard, different studies have shown that CAAs are able to secrete TNF-α, osteopontin, MMP9, and MMP11 [1,4,5,28]. These findings are related to CAAs involvement in the tumor extracellular matrix remodeling and in the increase in the metastatic abilities of the tumoral cells.

All things considered, CAAs mediate the CRC development and metastasis by induction of an immunosuppressive TME due to a large spectrum of adipokines, cytokines, and other molecular factors.

### 5.2. CAAs Metabolites in CRC

**Figure 4:**
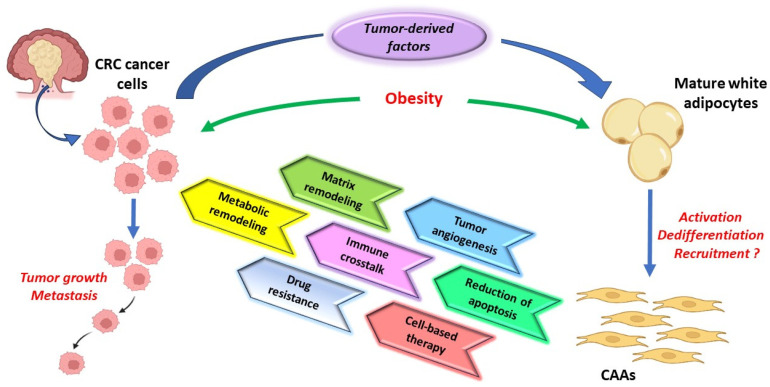
The relationship between tumor cells and CAAs in CRC tumor microenvironment. Adipocytes disposed at the tumor invasion front undergo lipolysis and transform into CAAs. CAAs support tumor growth and metastasis through a metabolic remodeling of cancer cells, local immune response modulation, tumor angiogenesis, and tumor extracellular matrix remodeling. Their activity is associated with resistance to therapy. Additionally, CAAs are cells that may be used to deliver therapeutic drugs into CRC TME. CAAs—cancer-associated adipocytes; CRC—colorectal cancer; TME—tumor microenvironment.

The bidirectional CRC cells–CAAs signaling is responsible for the intercellular metabolic symbiosis, which is established in the TME [99]. CAAs enhance their tumor-induced lipolysis and, in consequence, release different metabolites into the TME, such as adenosine triphosphate (ATP), lactate, pyruvate, and glutamine, along with FFAs, and which, in turn, increase the oxidation of the fatty acids in colorectal tumor cells [5,28]. In addition, CRC cells are able to adapt to the TME hypoxic status by using these metabolites released by different cancer-associated cells, including CAAs (Figure 4). Hypoxia also promotes the acetate uptake and hypoxia-inducible factor 1 (HIF-1) activation in colorectal cancer cells, which induces the sterol regulatory-element binding protein-1 (SREBP-1) and fatty acid synthase (FASN) overexpression, enzymes involved in de novo lipid synthesis [100]. 

As a relevant metabolite released by CAAs in tumor niche, FFAs support the cancer cell development by increasing the reactive oxygen species (ROS) synthesis and by metabolic remodeling [101]. Cancer cells also possess the ability to produce FFAs from glutamine and glucose, through de novo lipogenesis. These also remove FFAs from the TME through LPL activation and FFAs receptors overexpression, such as FABPs and CD36 [102,103]. Recent studies have been shown that FABP4 overexpression promotes epithelial-mesenchymal transition (EMT) and tumor cell metastasis by increasing the FFAs content into colorectal cancer cells co-cultured with adipocytes [104,105]. The colorectal cancer cells also use FABP4 and FABP5, rather than CD36, to scavenge extracellular FFAs and intracellular lipids, in contrast to other cancers, such as melanoma or ovarian and prostate carcinomas [103]. In addition, the reduction in FABPs expression has been associated with tumor progression inhibition, while a high FABP4 expression is correlated with CRC metastasis and poor prognosis [100]. Due to their pivotal role in tumor progression, recent studies focus on FFAs synthesis inhibition in the TME, as a potential therapeutic strategy for CRC patients [5]. In this regard, inhibitors of the FFAs supply to tumor cells, from endogenous and CAAs sources, may be used to treat patients with advanced forms of CRC, which do not respond to immune-checkpoint immunotherapy. As part of metabolic remodeling in the TME, glutamine contributes to 5-FU chemotherapy resistance through the mammalian target of rapamycin (mTOR) activation [106]. In addition, glutamine has been shown to support the cancer cells growth in human colon cancer cell lines [107] and induces angiogenesis via CXCL2-VEGFA pathway activation (Table 1) [108]. Moreover, a high level of glutamine in adipose tissue of CRC patients with peritoneal carcinomatosis has been observed in recent research, supporting the potential role of glutamine in colorectal cancer cells metastasis [109].

### 5.3. CAAs Immunomodulatory Role in CRC

The immune cells associated with CAAs in the CRC microenvironment have a large spectrum, which includes lymphocytes, macrophages, dendritic cells, myeloid-derived suppressor cells (MDSCs), neutrophils, natural killer cells (NKs), and mucosal-associated invariant T (MAIT) [110]. During tumor progression, CAAs support CRC onco-inflammatory milieu through release of proinflammatory cytokines, including IL1-β, IL-6, IL-8, and TNF-α that modulate the innate and adaptive immune response (Figure 4) [28]. Moreover, leptin and other immunomodulatory metabolites released by CAAs mediate the differentiation of adaptive immune cells [28]. In this regard, considerable evidence showed that CAAs contribute to local neutrophils recruitment and mediate NKs activity by reprogramming their metabolism by FFAs, IL-1 and IL-8 release [5,111,112]. IL-6 and leptin produced by CAAs are also able to reduce the cytotoxicity of NK cells via JAK/STAT3 signaling modulation and to promote the TME MDSCs infiltration in CRC patients (Table 1) [48,113]. Moreover, CAAs support tumor cell growth through alternatively polarization of macrophages to M2-like phenotype and by PD1 overexpression on CD8+ Teff that subsequently suppresses the antitumor activity of CD8+ T cells [28]. PD-L1 expression in CAAs also reduces CD4+T cells activity through activation of glycolysis and fatty acid oxidation (FAO), in several types of cancers, including CRC [5,28].

Another component of the immune system in the CRC TME is represented by MAIT, a type of lymphocyte that highly expresses CD161 and an invariant Vα7.2 of the T-cell receptor (TCR) chain [110,114]. MAITs contribute to tissue homeostasis through local control of bacterial infections [110,114]. The potential role of MAIT in CRC is still under debate. Currently, it is considered that MAITs may support colorectal carcinogenesis through release of IL-17 or by overexpression of cytotoxic effector factors that modulate the antitumoral immunity [110]. Significant evidence demonstrates that CAAs and visceral adipose tissue influence MAITs activity and induce MAIT-derived IL-17 secretion in obese patients with CRC [110,115]. In this respect, several studies have shown a depletion of the MAITs number in the blood and an increased infiltration of MAIT cells in the TME, in the advanced stages of CRC [116,117]. Moreover, the aberrant activation of IL-23/IL-17 axis in obese patients with colon adenocarcinoma is considered to contribute to a favorable TME for cancer progression through several pathways, such as local recruitment of MDSCs, increased expression of Bcl-2 and Bcl-x antiapoptotic molecules in cancer cells or local VEGF release (Table 1) [110,118]. 

**Table 1:** Potential roles of CAAs in CRC progression and metastasis.

		Pathways/Key Gene/Molecules/Receptors	Function	References
Adipokines	Leptin	c-Jun, Akt, and JAK/STAT3 axis IL-6, MMPs, and TGF-β ↑	carcinogenesis metastasis	[5,14]
	Adiponectin	AMPK axis	inhibition of tumor cell proliferation, angiogenesis, and metastasis	[5,64,65]
	Apelin	tumor growth and migration pathways	lymph node and distant metastasis	[75]
	Gherlin	*gherlin* gene ↓	increase in TME inflammation	[58]
	Resistin	Toll-like receptor 4 ↑ IL-6 and TNF-α ↑	promotion of tumor angiogenesis and metastasis	[1,76]
	Visfatin	ERK1/2, p38 MAPK, PI3K/mTOR, JNK, and JAK/STAT axis visfatin/SDF-1/Akt axis	support of tumor cell proliferation and metastasis inhibition of 5-FU therapeutic effect	[79,80]
	Insulin	PI3K/Akt pathway	increase in the resistance to 5-FU and cycloheximide cytotoxicity inhibition of tumor cell proliferation and metastasis	[81,84]
	IFGs	Src (tyrosine-protein kinase)	carcinogenesis proliferation of tumor cells	[85,86]
	VEGF	*VEGF* gene	tumor angiogenesis and metastasis	[96]
	IL-6	STAT3 axis	support of tumor growth and metastasis	[5,88,89]
	TNF-α	NF-kB, Wnt/β-catenin, ERK1/2 axis	carcinogenesis and tumor cell migration	[90,91,92]
	PAI-1	PAI1–tPA axis	increase in TGF-β expression in colitis CRC carcinogenesis and metastasis (?)	[94]
	CCL2	*CCL2* gene	increase in TME macrophages infiltration and induction of tumor progression	[14]
	MMP9 and MMP11	*MMP* genes	induction of tumoral extracellular matrix remodeling and metastasis	[1,4,5,28]
Metabolites	Lactate Pyruvate ATP	fatty acids oxidation	tumor growth and metastasis	[5,28]
	FFAs	FFAs receptors (FABPs and CD36)	increase in ROS synthesis and fatty acids oxidation promotion of tumor growth and metastasis	[101,102,103]
	Glutamine	CXCL2-VEGFA axis	support of angiogenesis, tumor growth and metastasis	[108,109]
Immune cells activity modulation	IL-6 leptin	JAK/STAT3 axis	promotion of MDSCs infiltration reduction in NK cells cytotoxicity modulation of the innate and adaptive immune response	[48,113]
	FFAs	FFA uptake	induction of neutrophils recruitment	[5,111,112]
	FFAs Lactate Glycerin	metabolic remodeling VEGF	reduction in T cells antitumoral activity and dendritic cells activation tumor angiogenesis and metastasis	[5,28,113]
	PD-L1	glycolysis and FAO	reduction in CD4+ and CD8+ T cells activity induction of M2-like macrophages polarization promotion of tumor growth and metastasis	[5,28]
	MAIT-derived IL-17	IL-23/IL-17 axis	promotion of MDSCs recruitment increase in VEGF and Bcl-2 in TME and Bcl-x expression in tumor cells carcinogenesis and tumor growth	[110,118]

ATP—adenosine triphosphate; CAAs—cancer-associated adipocytes; CCL2—chemokine (C-C motif) ligand 2; CRC—colorectal cancer; IFGs—insulin-like growth factors; IL—interleukin; FAO—fatty acid oxidation; FABPs—fatty acid binding proteins; FFAs—free fatty acid; MAIT—mucosal-associated invariant T cell; MDSCs—myeloid-derived suppressor cells; MMP—matrix metalloproteinase; NK cells—natural killer cells; PAI-1—plasminogen activator inhibitor-1; PD-L1—programmed death-ligand 1; ROS—reactive oxygen species; TGF-β—tumor growth factor β; TME—tumor microenvironment; TNF-α—tumor necrosis factor α; VEGF—vascular endothelial growth factor; 5-FU—5-fluorouracil; ↑—increase; ↓—decrease; (?)—working hypothesis.

In addition, CAAs are involved in cancer growth and metastasis through metabolic reprogramming and cytokines interacting with immune cells in the CRC microenvironment. In this regard, FFAs, lactate and glycerin produced by CAAs in CRC tumor milieu, facilitate the lipid droplets accumulation in NK cells cytoplasm that impair their antitumoral activity and prevent the cytotoxic T lymphocytes and dendritic cells activation [5,28,113]. CAAs-derived lactate also supports tumor invasion and migration by stimulating the secretion of VEGF into the TME and increases the expression of M2 macrophage polarization markers, including Mgl1, Mgl2, Arg1, and Fizz1 [28]. 

In light of these features, CAAs are a main component of the TME, which interact with the infiltrating immune cells to enhance inflammation and cancer progression. They also release a variety of adipokines and metabolic factors that induce dysfunction of the antitumoral immune response in CRC patients.

### 5.4. CAAs and Potential Therapeutic Strategies in CRC: New Trends and Future Perspectives

Recently, preclinical and clinical studies have opened new perspectives in CRC therapy by targeting CAAs, the nontumor-associated cells into the TME, which release different metabolites that promote tumor progression and therapy resistance in CRC [119,120].

Radical surgery associated with chemotherapy is the conventional treatment in CRC cases, 5-FU being the most common used drug in the advanced stages of the disease. Several mechanisms are currently related to 5-FU chemoresistance and treatment failure in CRC, such as *Bcl-2* and *Bcl-xL* overexpression in cancer cells, *p53* gene mutations, 5-FU efflux induced by the increased expression of the ATP-binding cassette transporter, nucleoside metabolizing enzymes overexpression, along with mismatch repair system disruption [80]. Adipocytes and CAAs-derived visfatin have been shown to reduce the sensitivity to 5-FU through SDF-1/CXCR4 pathway activation (Table 2) [80]. This finding could lead to researchers focusing on visfatin inhibitor use in association with 5-FU to reduce chemotherapeutic drug resistance, mainly in inoperable CRC cases.

Considering the role of FFAs in CRC progression, new pharmacological therapies can be developed by targeting FASN, an enzyme responsible for FFAs synthesis, which has been demonstrated to be overexpressed in CRC [121]. Consistent data revealed that de novo lipogenesis supported by FASN in the TME, supplies tumor cells with energy, which enhances their rapid proliferation. FASN inhibition leads to a decrease in tumor progression through several mechanisms, such as alteration of plasma membrane structure, an increase in cancer cell apoptosis, decrease in palmitate synthesis, and inhibition of Wnt, Akt, and β-catenin oncogenic signaling pathways [122]. The first-generation of FASN inhibitors, such as Orlistat, C75, and cerulenin, were tested in preclinical studies in different types of cancers, such as mesothelioma, lung, breast, renal, and prostate cancers [5,121,123]. However, their antitumoral effect has been associated with several side effects, such as weight loss or anorexia [123]. The potential therapeutic effect of FASN inhibitors in CRC is very limited [121]. In this regard, only TVB-3664, a new generation of FASN inhibitors, has been tested with no significant toxicity in CRC patient-derived xenograft models [120]. According to the same study, TVB-3664 treatment has been associated with a decrease in the tumor size in 30% of cases, with reduction in FFAs and phospholipids synthesis and with alteration of AMPK, Akt, and Erk1/2 oncogenic axis [120]. Based on these preliminary results, FASN drugs inhibitors should be used in the future, in association with standard chemotherapy, for the treatment of CRC patients; more so because CAAs exhibit the ability to metabolize chemotherapeutic drugs, thus contributing to their bioavailability decrease and to the chemotherapeutic resistance development.

CAAs contribute to the proinflammatory profile of the TME and reduce the antitumoral effect of T cells. In this regard, other approaches have been investigated, such as directly targeting CAAs or inhibiting the CAAs-derived signals into the TME in CRC patients. An attractive strategy for cancer treatment is represented by the inhibition of CD36 fatty acid transport proteins, which has been shown to induce the antitumoral infiltrating effector T cells accumulation and depletion of Tregs in the TME [124]. Moreover, CD36 blockage induces an increase in the antitumoral effect of anti-PD-1 therapy meditated by T cells in the TME [124]. Additionally, CD36 has been demonstrated to be involved in intercellular adhesion modulation, angiogenesis, and antigen presentation control in the TME [5]. In the same direction, a recent study has shown that CD36 blocking leads to cancer progression inhibition by lipid droplet accumulation decrease and intracellular ROS production reduction [125]. According to other recent studies, cancer progression and metastasis are also promoted by the enhanced FFAs uptake and FFAs oxidation induced by the overexpression of CD36 in tumor cells [126,127]. Last but not least, CD36 increased expression is associated with the upregulation of survivin, a protein which has been demonstrated to support cancer cell progression and resistance to therapy [128,129]. 

Considering this accumulated data, although the mechanisms of CD36 overexpression in CRC cases are incompletely deciphered, CD36 inhibition could represent a new therapeutic target for tumor progression limitation.

PPAR-ɣ is a master regulator of adipocytes differentiation and their CAAs-like phenotype acquisition in CCR [5,130]. PPAR-ɣ regulates lipid and glucose metabolisms, supports tumor growth through induction of apoptosis of T cells, and modulates the expression of tumor suppressor genes, such as *PTEN* and *BRCA1* [5,130]. Consequently, targeting PPAR-ɣ could represent another new direction of therapy in oncologic patients. In this regard, a study performed by Takano et al. demonstrated that pioglitazone, a PPAR-ɣ antagonist, inhibits CRC liver metastasis via downregulation of COX-2 and cyclin D1 expression on HT-29 and SW480 colon cancer cell lines (Table 2) [131]. Similar data have been reported by Zurlo et al. in their research on HT-29 colon cancer cell lines [132]. According to their results, cladosporol B (a PPAR-ɣ antagonist) administration induces a robust inhibition of tumor cell proliferation by their G0/G1-phase arrest [132]. Considering these findings, PPAR-ɣ antagonists have been proposed to be used as a new candidate target for CRC progression reduction.

Different metabolites released into the TME may modulate colorectal cancer cell proliferation and promote their chemosensitivity. From these metabolites, significant amounts of lactate accumulate in the TME, this is being considered as another potential therapeutic approach in CRC for decreasing immunosuppression mediated by CAAs [5]. In addition, cancer cells promote the pH regulators’ overexpression to escape from cellular acidosis, such as monocarboxylate transporters (MCTs) that mediate the lactate export from tumor cells into the TME. MCT1 has been demonstrated to promote tumor cell metastasis in different types of malignancies, such as neck, breast, colon, and bladder cancers [133]. AZD3965 is a specific MCT1 inhibitor that induces cancer cells death [5]. Recently, AZD3965 has gone through a phase I clinical trial for lymphomas and solid tumors (NCT01791595) [133]. As a result, AZD3965 should be considered as a potential new therapeutic tool for different types of cancers, including CRC [133].

**Table 2:** Potential CAAs therapeutic approaches in CRC.

Potential Drugs	Target	Mechanism	Study Model	References
Adipocytes and CAAs-derived visfatin	5-FU chemoresistance ↑	SDF-1/CXCR4 pathway	DLD-1 and SW620 CRC cells and CRC patient tissue samples	[80]
FASN inhibitors (TVB-3664)	FASN activity ↓	AMPK, Akt, and Erk1/2 pathway	CRC patient-derived xenograft model	[120]
CD36 (fatty acid transport proteins) inhibitors	CD36 blockage	anti-PD-1 therapy effect ↑ T cells infiltration ↑ Tregs cells activity ↓ lipid droplet accumulation ↓ ROS production ↓ FFAs uptake and oxidation ↓ (?) survivin expression ↓	Cd36 fl/fl mice inoculated with MC38 CRC cells mice CD36 shRNA and patients’ tumor tissue samples C57BL/6J mice inoculated with HCT116 CRC cells and CRC patient tissue samples	[124,125,128,129]
PPAR-ɣ antagonist (pioglitazone, cladosporol B)	PPAR	COX-2 and cyclin D1 expression ↓	CB-17/lcr-scid/Jclw mice inoculated with HT-29 and SW480 CRC cells	[131,132]
MCT1 inhibitors (AZD3965) (?)	MCT1	modulate the lactate export from tumor cells	phase I tumor clinical trial	[133]
CAAs-derived leptin inhibitors (Doxil)	Ob-R	tumor growth inhibition	BALB/c mice bearing C26 murine carcinoma	[134]

CAAs—cancer-associated adipocytes; FASN—fatty acid synthase; FFA—free fatty acid; Ob-R—leptin receptor; MTC1—monocarboxylate transporters 1; PPARɣ—peroxisome proliferator-activated receptor; PPAR-ɣ—peroxisome proliferator-activated receptor gamma receptor; 5-FU—5-fluorouracil; ↑—increase; ↓—decrease; (?)—working hypothesis.

Leptin is another promising therapeutic target in the fight against CRC, a CAAs-derived adipokine that supports colorectal carcinogenesis and tumor progression. In this direction, tumor progression inhibition has been induced by targeting the leptin receptor (Ob-R) in BALB/c mice bearing C26 colon carcinoma after Doxil administration [134]. However, these preliminary data should be validated by future clinical studies.

Taken together, CAAs represent an interesting niche that should be further explored for CRC treatment improvement. However, several challenges should be overcome before CAA therapies would be extensively applied. In this regard, a better understanding of the signaling axis involved in CAAs differentiation and the exact modality of obesity promotion of cancer progression will contribute to future therapeutic tools development against CRC when it is associated with overweight or obesity [1]. Moreover, future research on CAAs-mediated therapies is necessary to decipher the relationship between colon cancer cell heterogeneity and CAAs [28]. 

In addition, adipocytes, CAAs, and ADSCs may be used in a “Trojan horse” strategy to deliver the therapeutic drugs into the CRC TME (Figure 4) [135]. In this direction, miRNAs, drug-loaded exosomes and nanoparticles are recent additional tools that can be adopted for adipose cells-mediated antitumor molecules delivery into the TME [135,136,137]. Moreover, the frequent presence of CAAs in the invasive CRC front indicates that these cells may be used as suitable vehicles for various cytokines to improve the effector immune cell infiltration in the TME and tumor-based immunotherapy [1,135]. However, there are several limits of adipocytes use as cell-based delivery platforms in CRC. The therapeutic effect is most probably dependent on the quality and number of engineered adipocytes injected in their tumor niche and their ability to take over communication with cancer cells [135]. The clinical safety and effectiveness of adipose cell-mediated antitumor molecules delivery into the TME remain significant challenges in CRC treatment. Nonetheless, cellular-drug delivery systems may reduce the systemic toxicity of the drug and may overcome drug resistance in the hypoxic CRC TME. 

The development of nanoparticles that can be orally administered is another area of study that may lead to increased therapy compliance of CRC patients. However, one of the most important challenges in this direction is the prevention of gastric drug degradation [137]. Last but not the least, computer-assisted drug delivery may provide a better cellular-based antitumor molecules delivery into the TME by addressing different issues regarding the temperature, pH, salt concentration, enzyme-dependent nanoparticles preparation, and other external stimuli [136,137]. 

Although significant steps have been made to develop new antitumor therapeutic tools in CRC patients, multidrug resistance remains one of the main obstacles, being associated with different factors, such as gene mutation, drug transporter efflux, signaling pathways, TME heterogeneity, and the complex interplay between CRC cancer cells and nontumoral cells that include CAAs.

## 6. Conclusions

Growing evidence supports that the TME is a complex and dynamic cellular and molecular network, adapted to support tumor development and progression. CAAs are one of the most relevant nontumor-associated cells in the CRC TME, which display an active phenotype and contribute to the metabolic reprogramming of cancer cells and promote the onco-inflammatory milieu, by releasing a spectrum of adipokines, proinflammatory cytokines, growth factors, and hormones. 

A better understanding of CAAs’ biology may lead to a novel area of research to discover new anticancer-targeted therapies in CRC. In the context of tumor heterogeneity and complexity of TME cellular interplay, relatively limited studies have been conducted until now on the multi-faceted role of CAAs in CRC. Thus, further studies are warranted to characterize the molecular mechanisms underlying the association between CAAs, tumor cells, and tumor-associated cells in the CRC TME.

## Data Availability

Not applicable.
